# Insidious Manifestations of Cutaneous T-cell Lymphoma

**DOI:** 10.5811/cpcem.1393

**Published:** 2023-12-20

**Authors:** Alexis L. Cates, Heather P. Kahn

**Affiliations:** * Ochsner Medical Center, Department of Emergency Medicine, New Orleans, Louisiana; † Ochsner Medical Center, Department of Hospital Medicine, New Orleans, Louisiana

**Keywords:** *rash*, *neoplasm*, *dermatology*

## Abstract

**Case Presentation:**

A 66-year-old gentleman presented with several months of a generalized pruritic skin eruption along his face, thorax, and extremities. Although he had been seen previously, no diagnosis was made until he presented to the emergency department (ED) with worsening lesions. The patient was ultimately diagnosed with cutaneous T-cell lymphoma.

**Discussion:**

Accurately diagnosing a rash in the ED is not always possible as more invasive studies may be needed. Emergency physicians can expedite these studies where there is a high suspicion for a diagnosis that may need urgent evaluation and management by specialists through hospital admission and appropriate consultations. The clinical images here are an example of a rare disease manifesting as a debilitating rash, requiring inpatient evaluation and management.

Population Health Research Capsule What do we already know about this clinical entity?

*Cutaneous T-cell lymphoma is insidious and may resemble other pathology such as eczema or cellulitis.*

What is the major impact of the images?

*Diffuse symptomology and inclusion of the face and periorbital region warrant hospital admission for further urgent investigations.*

How might this improve emergency medicine practice?

*Physicians should keep cutaneous manifestations of systemic illness like cutaneous T-cell lymphoma on their differential for rashes.*

## CASE PRESENTATION

A 66-year-old Black male with documented history of peptic ulcer disease presented to the emergency department (ED) with a generalized skin eruption for four months. Initially he was prescribed an oral steroid and antihistamine for a pruritic eruption along his arms and face. This emergency visit was prompted when the rash reappeared on his torso and insidiously progressed to his face, arms, and thighs. He denied mucosal or genital lesions, exposures, contacts with similar symptoms, prescribed or over-the-counter medications, or international or domestic travel. He did not exhibit systemic symptoms of infection.

**Image 1.**
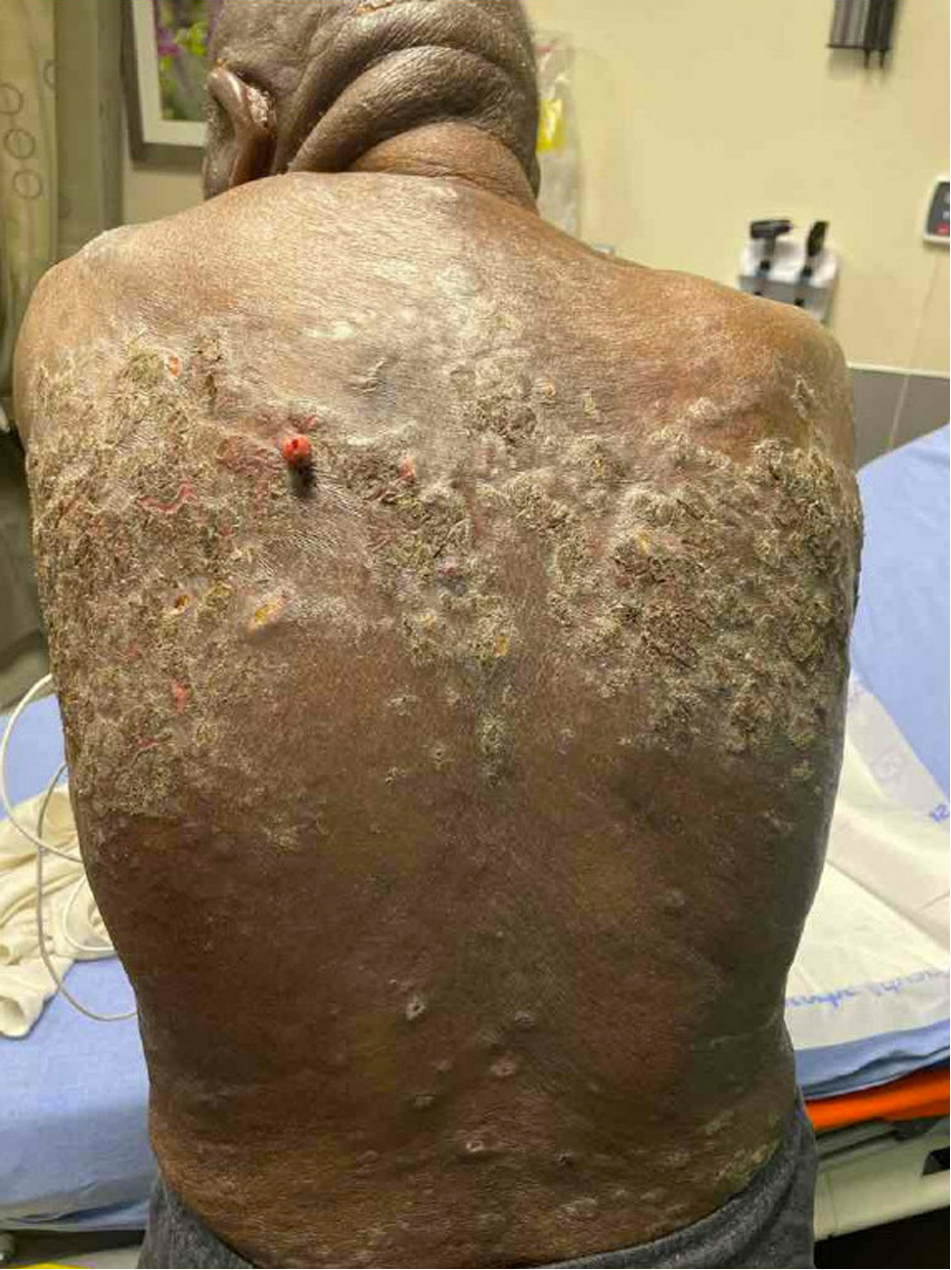
Scaly plaques with one large papular lesion near the left scapula in a patient with cutaneous T-cell lymphoma.

**Image 2.**
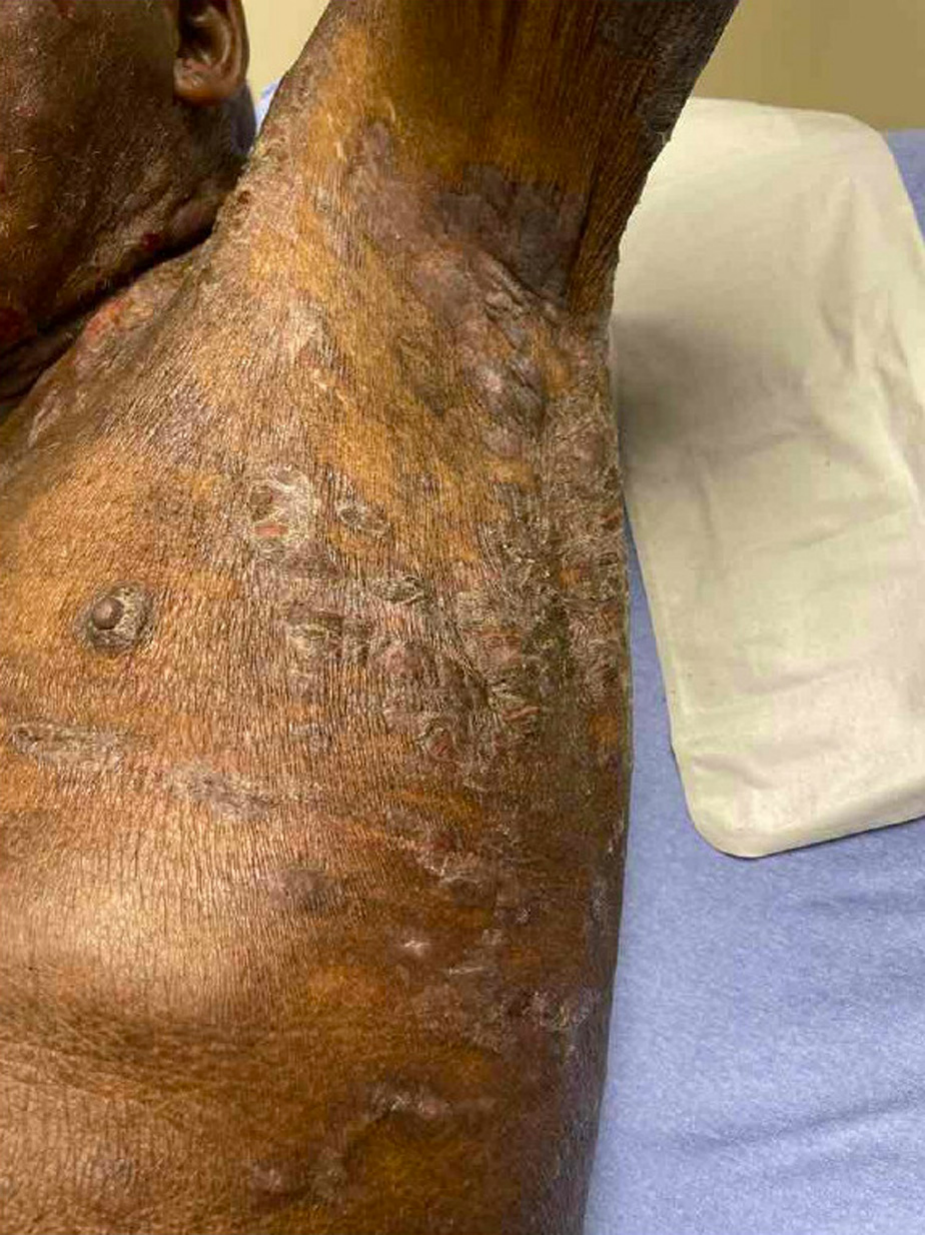
Scaly plaques secondary to cutaneous T-cell lymphoma along the patient’s left axilla and truncal region.

**Image 3.**
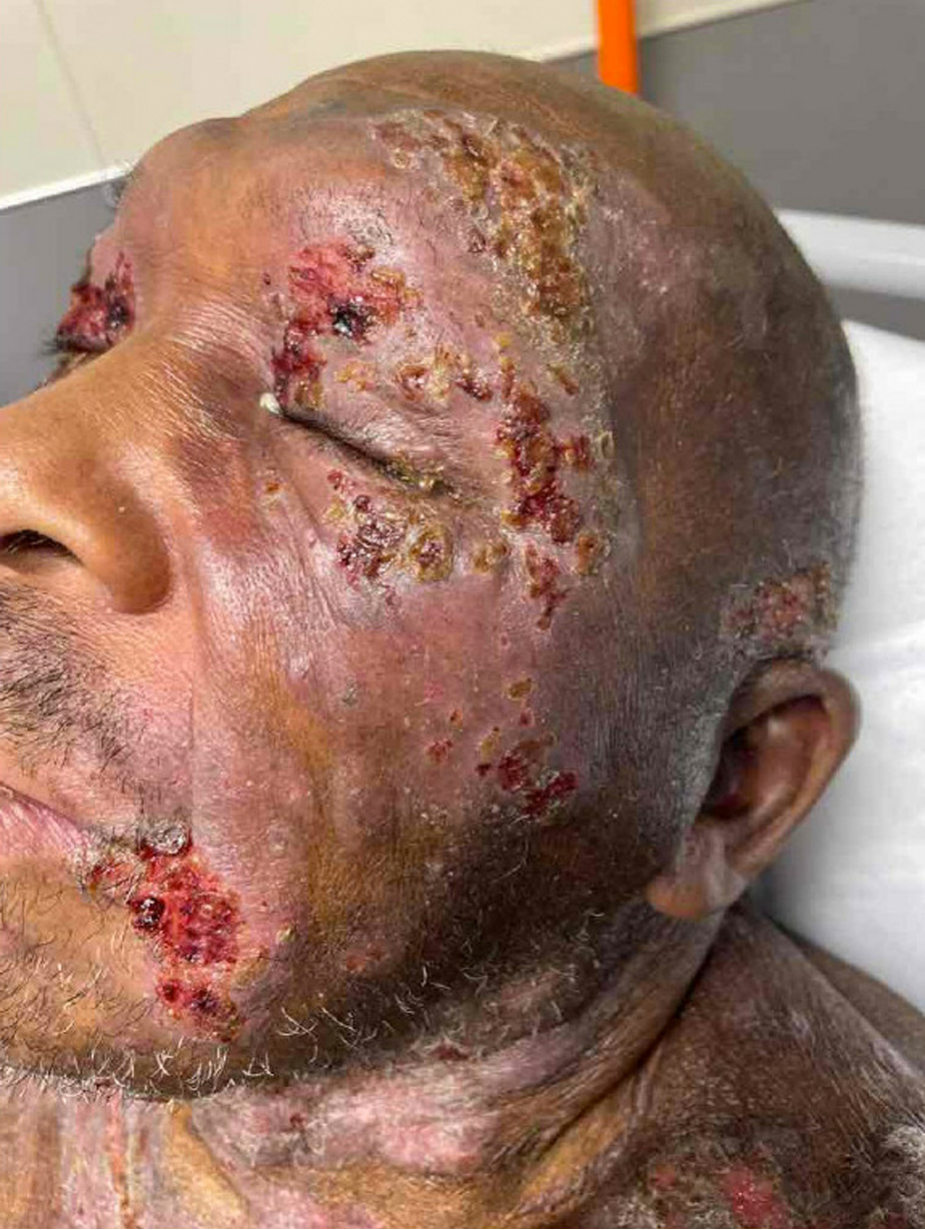
Crusted periorbital lesions, some of which were weeping and appeared to be superinfected, in a patient with cutaneous T-cell lymphoma.

The patient was well-appearing with a blood pressure 175/101 millimeters of mercury, pulse 77 beats per minute, and oral temperature 98.1° Fahrenheit. His skin revealed indurated and ulcerated plaque-like lesions with patchy erythema along the face and torso (Images 1 and 2). Plaque-like lesions were present on the thighs, and flat, scaly lesions were present on the hands and feet. There was no mucosal involvement. The periorbital lesions were vesicular and edematous but did not affect his vision (Image 3).

A computed tomography (CT) with iodinated contrast of the orbits showed preseptal and facial cellulitis. He was given clindamycin intravenously and oral hydroxyzine for itching. Pertinent labs included white blood cell count 5.8 × 10^9^ per liter (L) (reference range: 3.9–12.7 × 10^9^/L), hemoglobin 10.3 grams per deciliter (g/dL) (14.0−18.0 g/dL), platelets 200 × 10^9^/L (150–450 10^9^/L), international normalized ratio 1.0 (0.8–1.2), C-reactive protein 47.3 milligrams (mg)/dL (0.0–8.2 mg/L), erythrocyte sedimentation rate 128 millimeters per hour (mm/hr) (0–23 mm/hr) and normal electrolytes, renal function, and transaminases. A polymerase chain reaction test for herpes simplex virus was positive. Superficial aerobic cultures of the right chest and right back skin lesions resulted with methicillin-sensitive *Staphylococcus aureus.*

The patient was admitted to hospital medicine. Antibiotic coverage was broadened with vancomycin and ceftriaxone. Ophthalmology recommended topical erythromycin for the periorbital lesions. Dermatology raised concern for cutaneous T-cell lymphoma. A skin biopsy was obtained from the right back and sent to pathology for further diagnosis. Oncology recommended CT of the chest, abdomen, and pelvis. This revealed multiple areas of lymphadenopathy and mild splenomegaly suspicious for lymphoproliferative disorder. The patient declined an excisional lymph node biopsy. He was discharged from the hospital with a 10-day antibiotic course and outpatient oncology follow-up.

An outpatient positron emission tomography showed increased diffuse lymphadenopathy and soft tissue thickening, compatible with the suspected diagnosis of cutaneous lymphoma. Skin and right inguinal excisional lymph node biopsies confirmed T-cell lymphoma.

## DISCUSSION

Cutaneous T-cell lymphoma is a rare diagnosis with an incidence of approximately 8.55 per million according to a United States population data analysis from 2000–2018.^1^ Early in its course, it resembles eczema or psoriasis.^2^ It can take several years for the disease to progress to be diagnosed by biopsy.^2^ Most cases occur in patients 50–60 years old.^2^ Prognosis depends on lymph node and visceral involvement. Those with limited skin disease have a good prognosis.^2^ Treatment with topical or systemic immunotherapies depends on organ involvement.^2^ Morbidity and mortality may arise from infections of the ulcerated lesions. It is important for physicians to keep cutaneous manifestations of systemic illness like cutaneous T-cell lymphoma on their differential for rashes, particularly those that are chronic, disseminated, or debilitating.
